# Prospective evaluation of cardiac effects of first-time marathon training, running, and recovery in middle-aged men: cohort study rationale and design

**DOI:** 10.1007/s12471-022-01708-5

**Published:** 2022-07-14

**Authors:** I. Laily, T. G. H. Wiggers, N. van Steijn, E. Verhagen, A. J. Bakermans, H. T. Jorstad

**Affiliations:** 1 Department of Cardiology, Amsterdam University Medical Centers, University of Amsterdam, Amsterdam, The Netherlands; 2 Amsterdam Collaboration on Health & Safety in Sports, Department of Public and Occupational Health, Amsterdam Movement Sciences, Amsterdam University Medical Centers, Vrije Universiteit Amsterdam, Amsterdam, The Netherlands; 3 Center for Sport and Exercise Studies, Indonesian Medical Education and Research Institute, Faculty of Medicine, University of Indonesia, Jakarta, Indonesia; 4 Department of Sports Medicine, Anna Hospital, Geldrop, The Netherlands; 5 Department of Radiology and Nuclear Medicine, Amsterdam University Medical Centers, University of Amsterdam, Amsterdam, The Netherlands

**Keywords:** Endurance running, Myocardial remodelling, MRI, Echocardiography, Biomarkers

## Abstract

**Background:**

Several phenomena may point to potentially detrimental cardiac effects of endurance exercise, such as elevated circulating cardiac troponin levels and reductions in systolic and diastolic function directly after marathon completion. Furthermore, while myocardial abnormalities have been reported in patients who recovered from COVID-19, the cardiac impact of extensive endurance exercise in individuals who recovered from COVID-19 remains unknown. We therefore aim to investigate (potentially detrimental) cardiac effects of first-time marathon training and participation, including a subset of participants who recovered from COVID-19, in apparently healthy middle-aged men.

**Study design:**

This exploratory prospective cohort study investigates cardiac effects of first-time marathon running in 24 middle-aged (35–50 years) healthy men. Primary outcomes are cardiac morphological changes from pre-training up to 1 month after marathon completion, measured with magnetic resonance imaging (MRI) at 4 time points: 1) baseline (4 months before the marathon), 2) pre-marathon (2 weeks before the marathon), 3) post-marathon (< 24 h post-marathon), and 4) recovery (4 weeks after the marathon). Secondary parameters include other cardiac or non-cardiac changes: 1) quantitative MRI myocardial mapping, including mean diffusivity and extracellular volume fraction, 2) echocardiographic morphology and function changes, 3) VO_2_max, 4) electrocardiogram changes, and 5) levels of cardiac biomarkers.

**Discussion:**

This study will contribute to our understanding of cardiac adaptations and maladaptations to first-time marathon running in middle-aged men, and the interaction between extreme endurance exercise and potential detrimental cardiac effects, also in the context of COVID-19. Results will inform on future research directions while providing new clinical insights for health professionals involved in athlete care.

## Background

Regular participation in physical activity is associated with numerous health benefits, such as a reduced risk of fatal and non-fatal cardiovascular disease, improved aerobic capacity and strength, and a reduced body fat percentage [1, 2]. However, it is unclear what the upper limits of healthy physical exercise are and whether exceeding a certain amount of exercise may have detrimental effects on the cardiovascular system or general health [3].

Marathon running is a classic example of a popular sports activity that poses substantial demands on the cardiovascular, musculoskeletal, immune, metabolic, and neurological systems [4]. In the last three decades, the number of recreational marathon runners has markedly increased [5, 6], with middle-aged men constituting the largest group. Importantly, this is also the age group where atherosclerotic cardiovascular disease becomes clinically apparent [7].

Several phenomena that point to potentially detrimental cardiac effects of running have been reported, such as elevated biomarkers for myocardial injury (troponin T and I, NT-proBNP) [8, 9], and reductions in left and right ventricular systolic and diastolic function directly after marathon completion [10]. Most studies on cardiac effects of marathon running are cross-sectional or retrospective, do not include state-of-the-art imaging investigations such as cardiovascular magnetic resonance imaging (MRI) coupled with functional assessments, and do not include recovery follow-up. Furthermore, COVID-19 in athletes is associated with myocardial abnormalities, such as inflammation and fibrosis [11], but no studies have reported the effects of marathon running after recovery from COVID-19.

Our exploratory prospective cohort study in middle-aged men who train for and participate in their first marathon, including a subset of participants who recovered from COVID-19, aims to comprehensively investigate potentially detrimental cardiac changes during marathon training and participation.

## Methods

**Fig. 1:**
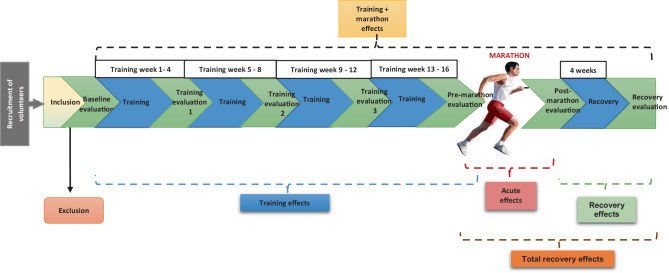
Study design diagram, with recruitment of volunteers, inclusion, and evaluations at multiple time points, to prospectively investigate the cardiac effects of first-time marathon training, running, and recovery in healthy middle-aged men. See Fig. 2 for details on evaluation parameters per study visit

This is an exploratory prospective cohort study in middle-aged first-time marathon runners. The study has been approved by the Amsterdam University Medical Centers Medical Ethics Review Committee (NL70800.029.19), and will follow the Declaration of Helsinki and adhere to the CONSORT guidelines [12]. Recruitment starts 6 months before the 2021 Amsterdam Marathon (Fig. 1).

## Outcomes measures

The primary outcomes are cardiac morphological changes from before initiating marathon training up to 1 month after completion of the marathon: 1) 4 months before the marathon, 2) pre-marathon (2 weeks before the marathon), 3) post-marathon (< 24 h post-marathon), and 4) recovery (4 weeks after the marathon).

Secondary outcomes include changes in other cardiac and non-cardiac parameters: 1) quantitative parameter mapping of the myocardium with MRI, 2) cardiac morphology and function with echocardiography, 3) VO_2_max, 4) electrocardiogram (ECG) characteristics, and 5) circulating cardiac biomarkers: high-sensitivity troponin T (hs-TnT), high-sensitivity troponin I (hs-TnI), and N‑terminal pro-B-type natriuretic peptide (NT-proBNP).

## Study population

Middle-aged (35–50 years) healthy men aiming to run their first marathon are eligible for our study. A lack of prior information on potential detrimental cardiac changes in such a cohort and the limited capacity to perform cardiac MRI examinations directly after the marathon requires choosing a convenience sample of 18 subjects. With an expected drop-out rate of up to 25% [13, 14], we aim to include 24 subjects. If possible, up to 50% of our study sample will have a history of COVID-19 (SARS-CoV‑2 infection without a history of hospitalisation). Table 1 presents all inclusion and exclusion criteria.

**Table 1:** Inclusion and exclusion criteria

*Inclusion criteria:*	
1	Man aged 35–50 years
2	No known current (uncontrolled) illness
3	No history of surgery for the past 2 years
4	No current self-reported musculoskeletal injury
5	Never trained for or completed a marathon
6	Never ran > 21.1 km in a single race or training in the previous year
7	Never trained or competed on a semi-professional or professional level in endurance sports
8	Informed consent
*Exclusion criteria:*	
1	Classified as high risk (symptomatic, or known cardiovascular, pulmonary, renal, or metabolic disease) according to the American College of Sports Medicine (ACSM) guidelines [16]
2	Contraindications for MRI examination at 3 T
3	Estimated glomerular filtration rate < 30 mL/min
4	Illiterate or unable to provide written informed consent

*MRI* magnetic resonance imaging

## Data collection and follow-up

**Fig. 2:**
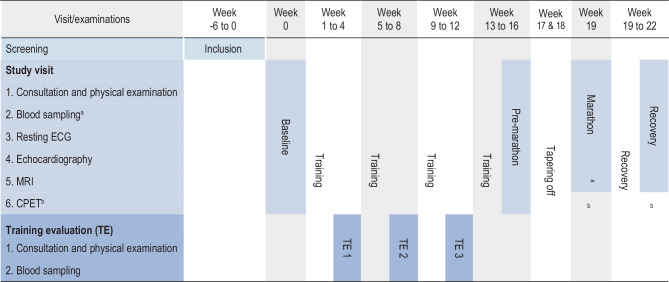
Evaluation parameters. ^a^ Blood sampling at three time points: before, within 2 h, and > 4 h after the marathon. ^b^ Cardiopulmonary exercise test (CPET) at baseline and pre-marathon visit only. *ECG* electrocardiography, *MRI* magnetic resonance imaging

All evaluations will be conducted at the Amsterdam University Medical Centers (Amsterdam, The Netherlands). Study and training evaluation visits are outlined in Fig. 2. Comprehensive evaluations will take place at four time points (i.e., study visits): 1) baseline (before initiating marathon training), 2) after 4 months of training (pre-marathon), 3) < 24 h after finishing the marathon, and 4) 1 month after the marathon (recovery). Evaluations at these visits will consist of a consultation and physical examination, blood sampling, ECG, echocardiogram, cardiac MRI, and cardiopulmonary exercise testing (CPET; at baseline and pre-marathon). Training history prior to inclusion will be collected and converted to metabolic equivalent of task hours per week (METh/week). Except for the immediate post-marathon study visit, subjects are advised not to engage in intensive physical activity/sports < 48 h before these visits. Training evaluation visits (3×) will occur monthly during the training phase (Fig. 1), and consist of training history, consultation, physical examination, and blood sampling. A licensed trainer will be available during unsupervised training for guidance. Subjective health will be assessed using the Dutch version of the Oslo Sports Trauma Research Centre (OSTRC) injury questionnaire [15].

## Inclusion

Potential participants will undergo screening prior to baseline according to the American College of Sports Medicine (ACSM) guidelines, which includes medical history and an evaluation of cardiovascular risk factors [16]. We will confirm that potential participants have never run a marathon before. If all inclusion criteria are met, and no exclusion criteria apply, participants will be included after signing informed consent. Subjects who are classified as high risk according to the ACSM guidelines at any time point during the course of the study will be excluded. If severe clinical and subclinical cardiovascular disease is found during the study, subjects will be asked to obtain referral for further cardiological or sports cardiological assessment.

## Consultation and physical examination

We will document the medical history and family history of cardiovascular disease, medication use, and the presence of cardiovascular risk factors according to the pre-participation ACSM guidelines [16]. Body mass, body height, waist circumference, resting heart rate, blood pressure, and auscultation abnormalities of the heart and lungs will be recorded.

## COVID-19

Our primary goal is to investigate cardiac changes through marathon training, running and recovery in first-time participants. We added COVID-19 status as a secondary, exploratory subgroup stratification. Subgroup of COVID-19 eligibility will be defined as: 1) history of any positive PCR test prior to baseline examinations without history of COVID-19-related hospitalisation, and/or 2) SARS-CoV‑2 antibodies in unvaccinated individuals at baseline.

## Blood sampling

Blood sampling will be performed according to standard procedures. Blood tests include a full blood count (FBC), glucose, lipid and renal profiles, electrolytes, creatine kinase MB (CK-MB), NT-proBNP, C‑reactive protein (CRP), hs-TnT, hs-TnI, and cardiotrophin‑1 (CT-1).

## Resting ECG

A standardised digital 12-lead resting ECG will be collected using the MAC 5000 ECG recorder (GE Healthcare, Chicago, USA).

## Echocardiography

Transthoracic echocardiography will be used to quantify systolic and diastolic function and chamber dimensions, and valve function. Imaging, measurements, and quantification will be performed according to the European Society of Cardiology recommendations [17].

## Magnetic resonance imaging

MRI examinations will be performed on three 3 T MR systems (Ingenia; Philips, Best, The Netherlands), and include functional cinematographic MRI and MR tagging, native T_1_ mapping, T_2_ mapping, diffusion tensor imaging (DTI), late gadolinium enhancement (LGE) MRI, and extracellular volume fraction mapping for an evaluation of myocardial tissue characteristics. Left and right ventricular volumes and myocardial mass will be quantified from cine MR images [18]. Myocardial strains will be estimated from cine and tagged MR images. LGE MRI will be performed approximately 10 min after intravenous administration of 0.4 mmol/kg body weight gadolinium dodecane tetra-acetic acid (Gd-DOTA, Dotarem; Guerbet, Villepinte, France) to assess focal myocardial fibrosis and the potential presence of scars. Subsequently, contrast-enhanced T_1_ mapping will be performed to estimate myocardial extracellular volume and diffuse fibrosis [18]. Concurrent haematocrit will be determined and used to correct extracellular volume estimates. Mean diffusivity and fractional anisotropy of myocardial tissue water will be quantified from DTI to assess cardiomyocyte integrity [19]. Throughout the study, each participant will be scanned on the same MR system to minimise potential system-specific variability between subsequent examinations.

## Cardiopulmonary exercise testing

CPET will be carried out according to the modified Dubowy protocol [20] for treadmill ergometry (Vaillant; Lode BV, Groningen, The Netherlands). The protocol will be continued until the subject reports exhaustion. Spirometry, a 12-lead ECG, pulse oximetry, and blood pressures will be measured throughout the course of the test.

## Training and sleep logs

Participants will be given a training logbook to document their training activity (distance, time, and heart rate) and sleeping patterns. Subjects in possession of a runner’s watch are encouraged to use their watches during training and asked to transfer their data at study visits.

## Statistical methods

Our study has an exploratory objective. Analyses will establish whether changes occur in the outcome variables of interest and guide the generation of hypotheses for future research. Data will be checked for normality using Shapiro-Wilk tests. Continuous variables will be reported as means with standard deviations or medians with interquartile range, as appropriate. Categorical variables will be presented as proportions and compared by a binomial test, Fisher’s exact probability test, or a chi-squared homogeneity test. Comparisons within individuals will be performed using mixed-model regression analyses. If there is a significant time effect, differences between individual time points will be assessed with paired *t*-tests. Analyses of covariates will be performed, and data will be corrected when appropriate. A level of *P* < 0.05 is considered statistically significant. We will register all reasons for drop-out, and assess potential attrition bias through comparison of baseline characteristics (and follow-up data when present) of completers vs. drop-outs.

## Discussion

Our study aims to prospectively investigate cardiac effects of first-time marathon training, running, and recovery in healthy middle-aged men. In addition, we will perform an exploratory analysis on whether a history of COVID-19 has pronounced cardiac effects during or after marathon training and participation. We aim to longitudinally collect detailed multi-modal data from before initiating marathon training until after recovery from running the marathon. Our data will shed light on myocardial adaptation and potential maladaptation, contributing to filling the existing knowledge gaps on potentially detrimental cardiac effects of endurance exercise.

Prior marathon studies demonstrated several sources of intrinsic bias or were limited to conventional investigative procedures, such as biomarkers, ECG and echocardiography [8–10]. A common source of bias is the inclusion of (highly) experienced runners only, who complete a comprehensive, investigator-led training programme, with evident and/or treated cardiovascular risk factors, leading to strongly selected study populations. The largest group of first-time runners, and simultaneously the group at the age where cardiovascular disease first becomes clinically evident, is the group of men aged 35–50 years. Our study therefore aims to specifically investigate cardiac effects in these first-time runners, and will yield clinically relevant data for this large group of individuals. However, not including women in our study population will limit the generalisability of our results.

We recognise several challenges pertaining to our study. First, little is known about marathon participation after COVID-19. This particular group may pose a recruitment challenge, and may also be particularly vulnerable to complaints or suboptimal training progression due to COVID-19 associated deconditioning [21]. No reports are currently available to assist physicians in maximising safety in sports in this group. Our extensive baseline screening and training evaluation visits will enable us to detect emergent pathology or serious training issues promptly. Finally, musculoskeletal injuries are common [22] in first-time marathon runners. We have taken this into account in our sample size of 24 participants, allowing for a 25% drop-out for a final sample size of 18 subjects.

## Limitations

Some aspects of our study warrant consideration. First, our study focuses on middle-aged male first-time runners specifically, and our findings cannot be readily extrapolated to older or younger men, individuals with pre-existent cardiovascular disease, or women. Second, while our subjects will be in considerably longer follow-up than in most previous studies, long-term follow-up (i.e. years) may be desirable.

Third, with a sample size of 24 individuals and an estimated drop-out rate of 25%, there is a chance of selective drop-out that could impact our findings. The majority of drop-outs in marathon studies is related to musculoskeletal problems [22], and will therefore have a limited impact on our cardiac outcomes of interest. Yet, specific subgroups may hypothetically demonstrate a propensity to drop out, such as individuals who recovered from COVID-19 or with an unknown genetic predisposition to adverse cardiac remodelling, which could potentially introduce attrition bias. We will therefore register all reasons for drop-out and analyse data of completers vs. drop-outs to assess potential attrition bias.

Nutrition plays an important role in marathon performance, and can affect anthropometric indices, blood pressure, lipid profiles and other cardiometabolic parameters. While we do not assess dietary patterns, we will collect comprehensive data on relevant risk factors, such as body composition and weight, blood pressure (at rest and during CPET), and lipid profiles and glucose metabolism. However, the impact of any potential changes in diet on our primary outcomes of morphological and structural cardiac changes during marathon running and training will be limited.

Finally, individuals with a prior asymptomatic SARS-CoV‑2 infection cannot be distinguished from individuals with antibodies due to vaccination, and will be classified as non-COVID-19 in our study. While testing for non-spike protein antibodies in individuals with an mRNA vaccination could potentially elucidate this further, such testing is beyond the scope of our study. Moreover, it is currently unclear what the clinical consequences (if any) of asymptomatic SARS-CoV‑2 infection in vaccinated individuals are regarding physical performance, pulmonary function, or cardiac structure and function.

In conclusion, our study will contribute to our understanding of cardiac adaptation and maladaptation to first-time marathon running in middle-aged men, and the interaction between extreme endurance exercise and potential detrimental cardiac effects in general, and in COVID-19 specifically. Our results will be hypotheses-generating, informing on future research directions while providing new clinical insights for endurance athlete care.
